# Athlete profiles along grit, sport orientation and sport persistence based on a quantitative research on Hungarian athletes

**DOI:** 10.3389/fspor.2025.1594365

**Published:** 2025-05-09

**Authors:** Karolina Eszter Kovács

**Affiliations:** Department of Counselling, Developmental and School Psychology, Institute of Psychology, University of Debrecen, Debrecen, Hungary

**Keywords:** sport persistence, grit, sport orientation, athlete profiles, cluster analysis

## Abstract

**Introduction:**

Sport persistence, the sustained engagement in sporting activities, is influenced by a combination of intrinsic and extrinsic motivational factors. Understanding these factors is crucial for supporting long-term athlete commitment and preventing dropout. The study examines athlete profiles based on sport persistence, sport orientation, and grit, aiming to identify distinct clusters that reveal different psychological and sociodemographic characteristics.

**Methods:**

A survey was conducted among 1,105 young athletes (aged 14–25) from secondary and tertiary education institutions in Hungary. The sample included both competitive and recreational athletes. Data collection involved validated psychological measures, including the Sport Persistence Questionnaire, Short Grit Scale, Sport Orientation Questionnaire, beside a block of sociodemographic questions. Cluster analysis (K-Means clustering) was used to identify athlete profiles based on sport persistence, grit, and sport orientation.

**Results:**

Four distinct athlete profiles emerged: (1) Consistently persistent athletes (*n* = 363; high levels of sport persistence, grit, and sport orientation); (2) Athletes prone to dropout (*n* = 174; low level persistence, grit, and sport orientation); (3) Oriented lagging athletes (*n* = 387; moderate sport orientation but lower grit and persistence); (4) Disoriented persistent athletes (*n* = 180; high grit but low sport orientation). Sociodemographic factors, such as parental employment status and educational background, significantly influenced group membership.

**Discussion:**

These results indicate that gender, educational level, parental employment, and sport type significantly influence an athlete's likelihood of maintaining sport participation. The findings also suggest that competitive team sports support networks may contribute to sustained engagement, while university transitions and socioeconomic challenges can lead to a decline in sport persistence.

## 1 Introduction

Sustained and active sporting activity can be influenced by several factors, which determine the manner, frequency and type of sporting activity. Based on the general (1) and sport-adapted (2) ecological model, intrapersonal, interpersonal, environmental and cultural factors are all relevant.

**Figure 1:**
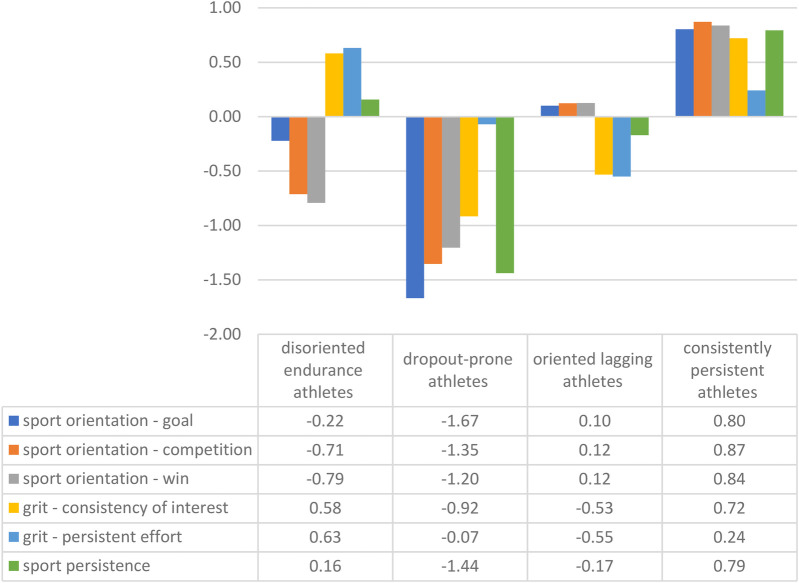
Characteristics of athlete profiles.

**Table 1:** Results of the multinominal regression analysis.

Model Coefficients—cluster membership					
Cluster membership	Predictor	Estimate	SE	Z	*p*
Dropout-prone athletes—disoriented endurance athletes	Intercept	0.37	0.28	1.32	0.19
	Gender				
	Male-female	0.67	0.25	2.72	0.01
	Sporting level				
	Competitive-recreational	−1.02	0.31	−3.29	<.001
	Level of education				
	Tertiary-secondary	−0.49	0.28	−1.75	0.08
	Sporting frequency				
	Every day—rarer than every day	−0.11	0.35	−0.32	0.75
Oriented lagging athletes—disoriented endurance athletes	Intercept	0.78	0.24	3.27	0.00
	Gender				
	Male-female	1.24	0.21	5.82	<.001
	Sporting level				
	Competitive-recreational	0.28	0.23	1.20	0.23
	Level of education				
	Tertiary-secondary	−0.98	0.23	−4.19	<.001
	Sporting frequency				
	Every day—rarer than every day	0.15	0.28	0.54	0.59
Consistently persistent athletes—disoriented endurance athletes	Intercept	−0.20	0.26	−0.76	0.45
	Gender				
	Male-female	0.97	0.22	4.41	<.001
	Sporting level				
	Competitive-recreational	1.21	0.24	5.10	<.001
	Level of education				
	Tertiary-secondary	−0.52	0.24	−2.14	0.03
	Sporting frequency				
	Every day—rarer than every day	0.78	0.27	2.86	0.00

Motivation is an umbrella concept, i.e., a multifaceted and complex phenomenon, and its examination can have several aspects, in each case it depends on the way the question is posed which aspect is considered (3). It is based on need, which is always linked to a state of deficiency. It may be physiological or higher, conscious or unconscious. The way in which the need is met is influenced by the environment, but also by our past experiences.

Beyond, but based on regular physical activity, commitment to sport refers to a higher level of engagement where a person builds on their strengths and overcomes their disadvantages by engaging in active sport (4). As with regular sport participation, motivation is the cornerstone of commitment and determines the extent and quality of engagement (5, 6). Scanlan and colleagues’ Sport Engagement Model (7–9) are considered authoritative in international practice. Their model has been modified and extended several times. In their later model, Scanlan et al. (9) based their model of sport engagement on the factors that determine sport participation mentioned above. Among the factors within the individual, enjoyment of sport as a basic segment is highlighted, alongside which internal resources and opportunities are emphasised. Other influencing factors may also be the opportunities given to the individual or arising for the individual, as well as other commitments that may distract the athlete from sporting activity, even leading to drop-out in the long term. It is the combination of these components that leads the athlete to decide on the intensity of sporting activity or on sport as a career. In a social context, the social support, including the role of family, peers and the sports club, should be mentioned. At the same time, peer pressure should not be left out, referring to societal expectations and norms (9, 10).

Commitment to sport is the basis for the main thrust of the research, called sport persistence that can be regarded as an indicator of effectiveness. It is a complex concept, a terminology used to express the mastery, form, level and effectiveness of sporting activity. However, its use is less widespread as theories tend to focus on sport motivation and commitment, which do not fully correspond to the concept of sport persistence, as it encapsulates the physical, mental and social aspects of sport (11). It goes beyond sport motivation and commitment; it is the embodiment of sport performance and mental toughness. Overall, sport persistence encompasses psychological skills such as resilience (11), adaptive and proactive coping (4, 10) and positive personality traits, which may also contribute to dropout prevention and enable athletes to show greater commitment and perseverance (12). This refers to the attempts to resolve, process and use stressful situations related to performance plateaus, failures, injuries or even successes and positive events. This behaviour and performance is captured by the concept of sport persistence, the study of which is not widespread in international practice, as research typically focuses on sporting habits, sport motivation and engagement (4, 13). Overall, sport persistence can be considered an outcome indicator, while it refers to a person's performance through sustained physical activity (regardless of the level of activity).

Research findings on sporting behaviour and engagement, both at national and international level and at sporting discipline level, suggest that the issue of sport persistence is worth addressing. Investigating the sporting habits, commitment to sport and persistence of student athletes at different educational levels is also crucial to understanding the balance between the field and the role of the athlete (14). For this reason, investigating the dual career model of student athletes is key, especially for competitive athletes. On the one hand, we need to recognise the unique challenges faced by young people who aspire to perform at a high level in both academic and athletic fields. This highlights the complex balance required between athletic behaviour and academic aspirations (15). Understanding this model will help to design tailored support systems to ensure that student athletes can thrive in both areas without sacrificing one for the other. In addition, exploring this concept will help to optimise training schedules, academic pathways and career transition strategies, ultimately improving the holistic development and well-being of student athletes as they navigate the pillars of their dual careers.

The study aims to identify athlete profiles based on sport persistence, sport orientation, and grit. During the research, we had the following assumption: *It is assumed that distinct athlete profiles can be created along the lines of sport persistence, sport orientation and grit, which are characterised by different sociodemographic and athlete, individual and social psychological characteristics.* It investigates the psychological and sociodemographic characteristics that influence sport persistence and examines how factors such as gender, education level, parental employment, and sport type contribute to an athlete's likelihood of maintaining long-term engagement in sports.

## 2 Methods

### 2.1 Sample

A total of 1,209 completions were received, but the data cleaning (based on the inclusion and exclusion criteria) allowed the analysis of data from 1,105 respondents. Inclusion criteria were established as follows: (1) participation in sporting activities on a regular basis (at least once a month), regardless of the type (individual or team) or level (recreational or competitive) of the activity; (2) enrollment in secondary or higher education; and (3) age ranging from 14 to 25 years. Individuals younger than 14 years, older than 25 years, or those not currently engaged in secondary or tertiary education were excluded from the study.

To investigate secondary school students, the primary aim was to include students from grades 9–13 learning in educational sports schools, as these institutions offer an ideal environment for examining both competitive and recreational athletes. The sports school program serves a dual purpose: it fosters both athletic and academic pursuits, thereby offering suitable opportunities for adolescents engaged in higher-level sports (16). Furthermore, prior studies have indicated that a considerable number of students enrolled in sports schools are primarily recreational athletes rather than competitive ones. Additionally, sports schools generally feature various types of classes (i.e., different profiles), which makes educational sports schools a fitting context for the development of recreational athletes to serve as a control group. In the context of higher education institutions, it is not feasible to continue this line of sports schools, as currently, the only establishment that can be classified as an educational sports school in Hungary is the Hungarian University of Sports Science. However, the presence of only one higher education institution would not suffice for a comprehensive study. Consequently, the integration of students engaged in competitive sports within higher education institutions was intended to be facilitated through university sports clubs. Finally, the combination of institutional outreach and snowball sampling was applied to maximise participation, while acknowledging the limitation that convenience sampling may still introduce some selection bias. In our study, snowball sampling was considered as a non-random sampling technique in which initial participants refer new subjects to the study (17). This method is particularly useful for studying hard-to-reach or special populations where a complete list of potential participants is not available. Initial participants were identified and contacted by age and sports school membership. They referred additional participants who later joined the study. This chain reaction of recruitment continued until the desired number of elements was reached.

Data collection took place between 8 January 2024 and 20 June 2024. The basic concept of the sample design was organised around the secondary school and university student population, and therefore, our sample presentation follows primarily this distribution. 43.3% of participants studied secondary education, and 56.7% studied tertiary education.

Regarding gender, 52.3% of the sample are female and 46.9% male, while 0.8% of the respondents did not specify their gender. Regarding the frequency of participation in sports, almost half of the sample (49.5%) participates in sports several times a week. The proportions of daily (18.5%) and several times a day (14.8%) exercisers are also relatively high, followed by those exercising once a week (9.3%) and monthly (7.9%).

The proportion of individual athletes is higher (57.5%) compared to team athletes (42.5%). The differences in distribution are also significant for the two subsamples in this case. The proportion of sports club members and non-members is almost equal, with 49.5% of the former and 50.5% of the latter.

Regarding the sporting level, the highest proportion of recreational athletes is 51.8%, which implies that 48.2% of the sample can be considered competitive athletes. Of these, 28.2% regularly take part in national championships and cups, 9.2% in county championships, 5.7% in international competitions and 5.1% in local and city competitions.

### 2.2 Instruments

The research was conducted using validated questionnaires and questionnaires under validation. Accordingly, the questionnaire consists of the following units: a socio-demographic questionnaire, a block of sport and health-specific questions, a block of questions related to academic performance, psychological measures (perception of success, sport orientation, sport persistence, sports anxiety, grit, resilience, well-being, vision, peer support, perfectionist climate, human values), and questions on relational embeddedness. While several psychological constructs were measured during the study, we selected grit, sport orientation, and sport persistence for analysis because these constructs were most theoretically relevant to our research questions. Therefore, we used only these psychological variables as independent ones in our analyis.

#### 2.2.1 Socio-demographic questionnaire

At the beginning of our questionnaire, various socio-demographic questions were registered: gender, age, current level of education (secondary or higher) and grade, type of settlement, parents’ educational attainment, parents’ employment, and objective financial situation.

#### 2.2.2 Sport-and health-specific issues

After the socio-demographic data, sport-specific data were queried: self-rated health (10-point Likert scale), self-assessed fitness status (10-point Likert scale), frequency of exercise (several times a day, daily, several times a week, once a week, monthly, less often), sporting activity in an association (yes/no), weekly time spent in sports club training (hours), weekly time spent on individual training (hours), type of sport (individual, team), sporting level (international competitions, national championships, county-level championships, local championships, non-competitive)

#### 2.2.3 Short Version Sport Orientation Questionnaire

The questionnaire consists of 13 items and includes three subscales: win orientation, goal orientation and competition. Win orientation measures the willingness to win, and goal orientation focuses on sports goals through winning the game (17). Competition, on the other hand, measures competitive orientation through anticipation of the game, enjoyment of the game and achievement of the goal of the game. The statements are rated by the respondents on a 5-point Likert scale. The validity of the measure was adequate for all subscales (Cronbach's α = 0.86; 0.85 and 0.89).

#### 2.2.4 Sport Persistence Questionnaire

The Sport Persistence Questionnaire (19) is a 13-item questionnaire that measures sport persistence through a factor. Items are rated on a 5-point Likert scale. The reliability measured in the original survey is Cronbach's α = 0.943. The minimum score on the overall index is 14 points, and the maximum score is 65 points. A higher score indicates higher persistence.

#### 2.2.5 Short Grit Scale

The instrument is designed to measure grit, a combination of passion and perseverance, through a single-factor question structure consisting of eight statements (20). The average of the scores obtained is used as the basis for the assessment. The items are interpreted on a 5-point Likert scale. The questionnaire also contains four reverse items. The measure includes two subscales: consistency of interest (Cronbach α = 0.77) and perseverance of effort (Cronbach α = 0.82).

### 2.3 Statistical analysis

The data was collected in an Excel spreadsheet. Data analysis was performed using IBM SPSS 22.0 and Jamovi 2.3.28 statistical software. The distribution of the data was examined using normality tests, such as Kolmogorov–Smirnov and Shapiro–Wilk tests. The data follow a non-normal distribution (*p* < 0.05). To create student profiles, cluster analysis (K-Means cluster, Iteration: 100) was used including the following variables: sport persistence, grit (persistent effort and consistency of interest), and sport orientation (competition, goal and win orientation).

## 3 Results

Since these scales do not have the same score, the clustering of athletes was done after standardization of the variables. The results showed that the athletes in the sample could be classified into four clusters (Figure 1). To determine the optimal number of clusters, we evaluated internal validation metrics, including the Silhouette Score and the Elbow Method. The silhouette analysis revealed that the optimal cluster count was k = 4, which maximized inter-cluster separation and intra-cluster cohesion. The elbow plot showed a marked inflection at k = 4, supporting this choice. These results suggest that the selected number of clusters represents a stable and well-separated grouping structure in the data.

The *consistently persistent athletes* (N_consistently persistent_ = 363) cluster included those with high grits and sport persistence, and significantly above average scores for different types of sport orientation. The opposite pole was the group of *dropout-prone athletes* (N_dropout−prone athletes_ = 174), with low values for both general and sport-specific persistence and below average values for sport orientation. In addition to the two “extreme” groups, two clusters with mixed patterns were formed. The cluster of *oriented lagging athletes* (N_oriented lagging athletes_ = 387) consisted of individuals with slightly positive, i.e., slightly above average, levels of goal, victory and competition orientation, but with below average grit and sport persistence scores. Finally, for *disoriented endurance athletes* (N_disoriented persistent athletes_ = 180), none of the types of sport orientation are present and sport persistence is only slightly above average, while overall persistence and grit are relatively high.

A significant difference in the distribution of group profiles along gender was observed (*χ*^2^ = 77.152; df = 3; *p* < 0.001). Women were overrepresented in the disoriented persistent and dropout-prone groups, while men were overrepresented in the oriented lagging and consistently persistent groups of athletes (Supplementary Table S1).

Significant distributional differences were also observed with respect to level of study (*χ*^2^ = 189.550; df = 3; *p* < 0.001; Fisher exact test = <0.001; Cramer's V = 0.261; Kendall's Tau-B = 0.210). Consistently persistent athletes were clearly over-represented among high school athletes. In contrast, disoriented persistent athletes and dropout-prone athletes are found in significantly higher proportions among secondary school students (Supplementary Table S2).

There are also significant distributional differences in the labour market status of the father across athlete clusters (*χ*^2^ = 24.952; df = 3; *p* < 0.001; Fisher exact test = <0.001; Cramer's V = 0.277; Kendall's Tau-B = −0.231). Among consistently persistent athletes, athletes whose father is employed are overrepresented, while among disoriented persistent and dropout-prone athletes, athletes whose father is not employed are overrepresented (Supplementary Table S3).

Concerning mother's education (*χ*^2^ = 8.382; df = 6; *p* = 0.211) and father's education (*χ*^2^ = 6.471; df = 6; *p* = 0.373), mother's employment (*χ*^2^ = 2.329; df = 3; *p* = 0.507), change in family structure (*χ*^2^ = 3.903; df = 3; *p* = 0.272), having a sibling (*χ*^2^ = 6.560; df = 3; *p* = 0.087), no significant difference was detected.

There were also significant differences in distribution along sporting frequency. Consistently persistent athletes were over-represented among those who participated in sport daily or more than once a week and under-represented among those who participated in sport less than this. Among dropout-prone athletes, there is an over-representation of those who participate in sport several times a week, monthly and less frequently, while those who participate in sport daily or several times a day are markedly under-represented in this group. For disoriented persistent athletes, the representation of weekly athletes was detectable, while athletes who exercise daily or several times a day and those who exercise monthly or less frequently were underrepresented. Neither over- nor under-represented cluster membership rates were found for oriented lagging athletes (Supplementary Table S4; Cramer's V = 0.260; Kendall's Tau-B = 0.221).

In terms of the type of sport (Supplementary Table S5), it can be seen that team athletes are over-represented among consistently persistent athletes and are also more prevalent in the oriented lagging athlete cluster. In contrast, individual athletes are overrepresented in the disoriented persistent and dropout-prone athlete clusters. The difference in distribution is also significant in this case (*χ*^2^ = 95.281; df = 3; *p* < 0.001; Fisher exact test = <0.001; Cramer's V = 0.294; Kendall's Tau-B = −0.233).

A significant difference in the distribution of cluster memberships was also found with respect to sporting level (*χ*^2^ = 189.550; df = 3; *p* < 0.001; Fisher exact test = <0.001; Cramer's V = 0.414; Kendall's Tau-B = 0.358). The consistently persistent athlete cluster showed a clear overrepresentation of competitive athletes, while the disoriented persistent athlete cluster and the disoriented recreational athletes were also overrepresented (Supplementary Table S6).

Finally, significant distributional differences were also found for sport-specific variables along association membership (*χ*^2^ = 142.529; df = 3; *p* < 0.001; Fisher exact test = <0.001; Cramer's V = 0.359; Kendall's Tau-B = −0.309). Among consistently persistent athletes, young people playing sport in sports associations were over-represented, while among disoriented persistent and dropout-prone athletes, those pursuing sport without sports association membership were significantly over-represented (Supplementary Table S7).

To assess the variables influencing cluster membership, multinominal regression has been performed (Table 1). Gender was a consistent and significant predictor in all models, with males being more likely than females to belong to dropout-prone, oriented lagging and consistently persistent athletes compared to disoriented endurance athletes. Sporting level was also a significant factor; individuals engaged in competitive sports were less likely to be in the cluster of dropout-prone athletes (*p* = 0.001) but more likely to be in the cluster of consistently persistent athletes (*p* = 0.001), compared to those involved in recreational sports. Education level showed a significant negative association with the cluster of oriented lagging athletes (*p* = .001) and that of consistently persistent athletes (p =0.03), indicating that individuals with tertiary education were less likely to be in these clusters compared to those with secondary education. Lastly, sporting frequency significantly predicted membership in the cluster of consistently persistent athletes (*p* = .00), with those who exercise daily more likely to belong to this group than those with less frequent activity. These results suggest that gender, sporting level, education, and frequency all play roles in determining cluster affiliation. The model shows statistical significance and acceptable (though modest) explanatory power (AIC = 2,600; BIC = 2,705; R²McF = 0.100; R²N *χ* = 0.132; *χ*² = 289; df = 12; *p* < 0.001).

## 4 Discussion

In this research, four athlete profiles were identified using cluster analysis based on the variables of athlete persistence, grit and sport orientation. *Consistently persistent athletes* were characterized by high sport persistence and grit, and above average sport orientation scores. Such athletes have a deep intrinsic motivation that helps them to achieve long-term goals. They are able to clearly define their goals and work continuously to achieve them. *Dropout-prone athletes* have low levels of both general and sport-specific persistence and sport orientation. They find it difficult to stick with sport in the long term. They often lack clear goals and orientations in sport. They are less persistent and determined, easily giving up sport when faced with difficulties. *Oriented lagging athletes have* a slightly above average goal, winning and competitive orientation, but their grit and sport persistence scores are below average. While they have the desire to compete and win, they lack the perseverance and determination to keep participating. They lack the internal resources, such as grit and sports persistence, needed for long-term commitment. Finally, *disoriented endurance athletes* are characterised by relatively high overall endurance but below average sport orientation and sport persistence. They are persistent and determined in everyday life, but less motivated to participate in sport and less committed to sport. They may not have found a sport in which they are truly committed and motivated, or they may have taken up sport under external pressure or expectations but not find intrinsic motivation and enjoyment.

In terms of gender differences, women are overrepresented in the disoriented persistent and dropout-prone groups, while men are overrepresented in the oriented lagging and consistently persistent groups. This finding is consistent with the research finding of typically high sport persistence among men, and further research has highlighted higher levels of grit and sport orientation among men (21, 22). For girls, social expectations often lead to less incentive for competitive attitudes and persistence in sport, which may result in lower sport persistence (23).

In terms of level of study, it can be seen that consistently persistent athletes are over-represented among high school athletes, while disoriented persistent athletes and drop-out prone athletes are over-represented among tertiary students. This trend is not surprising in light of our research finding that high school athletes have significantly higher levels of sport persistence compared to university athletes. This may be due to a number of factors, such as the career potential of secondary school students or the fewer responsibilities in secondary school students’ lives (e.g., work, family), which may result in more time and energy for sport (24, 25). In comparison, university students’ schedules are often less structured and require more autonomy, which may make it difficult to maintain regular sport participation. In addition, university students often have more responsibilities (e.g., study commitments, work, financial burdens), which can reduce the time and energy available for sport (24, 25).

In terms of sociodemographic factors, a significant distributional difference in athlete profiles was found based on the labour market status of the father, with a higher proportion of athletes with a working father/carer among consistently persistent athletes, while children of non-working fathers were over-represented among disoriented persistent and drop-out-prone athletes. Sporting activities often involve significant costs (e.g., equipment, coaching fees, travel). Working parents are more likely to be able to provide these financial resources, which allows their children to continue playing sport (26). Financial security reduces stress on families, which can have a positive impact on children's overall wellbeing and allow them to focus on sport (27). In addition, working parents often serve as strong role models for their children in terms of perseverance, responsibility and work ethic, which can have a positive impact on children's sporting habits and perseverance (19).

There was also a significant difference in the distribution of sporting frequency. Among consistently persistent athletes, daily or multiple daily sporting activity is a characteristic, which is logical given that high intensity sport is a straightforward contributor to sport persistence (28, 29). For them, sport is not just a hobby, but a lifestyle and a passion. These athletes often have specific sporting goals, such as preparing for competitions, improving performance or maintaining health, which motivate them to train regularly. Athletes at risk of dropping out are more likely to participate in sport on a monthly or less frequent basis, which was also a previously hypothesised finding given that athletes at risk of dropping out typically have lower sporting intensity (30). Athletes at risk of dropping out often struggle with a lack of motivation, which contributes to lower sporting frequency. For these athletes, playing sport is not *a priori*ty and other activities such as studying, work or social life take precedence over sport in their schedule (31). Among disoriented endurance athletes, weekly athletes are in the majority. These types of athletes have a certain level of commitment to sport, but it is not as intense as seen in their consistently persistent counterparts. For them, playing sport is important but not *a priori*ty. The frequency of weekly sporting activities indicates that they try to find a balance between sport, work/study and social life. Although not as intensively as the consistently persistent sportsmen and women, they try to include sport regularly in their lives.

There were also significant differences by sport type. Team athletes were overrepresented in the consistently persistent and oriented dropout groups, while individual athletes were overrepresented in the disoriented persistent and dropout-prone groups. This distribution reflects our research findings that team athletes are characterized by higher sport persistence. In team sports, players strive for common goals, such as winning a championship or improving team performance. These shared goals increase the level of grit, as athletes are willing to persevere over the long term to achieve these goals (21). In addition, this trend confirms the findings of previous research that team athletes are also considered to be more effective in terms of grit as well as sport orientation (21, 32).

In terms of sporting level, there is a clear over-representation of competitive athletes in the consistently persistent group, while recreational athletes are significantly over-represented in the dropout-prone and disoriented persistent groups. Competitive athletes often set high goals for themselves, such as winning competitions, championships or national level success. These goals increase commitment and persistence in sport. In addition, competitive athletes often compete not only for themselves but also for their team or association, which further increases motivation and commitment (26, 33). In contrast, the main motivation for recreational athletes is often for fun, health maintenance or social interaction. These goals are less powerful than those in competitive sports and do not necessarily require ongoing commitment (34). In addition, recreational athletes are more flexible in managing their sporting habits, which can make them less consistent and persistent (35).

Differences by sports club membership show an overrepresentation of athletes pursuing sport in sports associations in the consistently persistent group, while athletes who do not pursue sport in sports associations have a significantly higher proportion of disoriented persistent and dropout-prone groups. This is consistent with our research findings that members of sports clubs have higher sport persistence. In clubs, athletes often find themselves in a motivating environment where they can be inspired by the success of others, which can also be reflected in higher levels of competitive participation (36).

The novelty of our study lies in the identification and typology of athlete profiles through a multivariable cluster analysis based on grit, sport orientation, and sport persistence, a combination that has not been previously used in this population. The emergence of two mixed-profile types—oriented lagging athletes and disoriented persistent athletes—is particularly noteworthy. These groups reflect psychological inconsistencies, such as high orientation but low grit (or vice versa), which have not been distinctly captured in prior literature. This offers a nuanced view of athlete motivation and engagement beyond high or low persistence categorisations. Moreover, the study also highlights how sociodemographic and contextual factors (e.g., parental employment, educational level, club membership) systematically relate to psychological profiles. This has important practical implications for targeted interventions in sport psychology and athlete development, especially in the Hungarian context, where such large-scale data has been previously lacking.

Among the limitations of the research, the limitations of the sampling method should be highlighted. The originally planned two-stage stratified sampling was not feasible as most institutions refused to participate in the research. For this reason, snowball sampling was used, but this may distort the results and reduce their generalisability. In addition, it should be mentioned that some questionnaires are not considered as validated instruments in the country, and the primary objective of the research itself was not to validate the instruments, but the study sample allows for the domestic validation of non-validated questionnaires, which could be a further research direction. For some of the insignificant effects, it is suggested that the use of other instruments may be justified to measure certain influencing factors (e.g., peer support, social values). Furthermore, explanatory variables related to meso- and macrosystem levels were underrepresented in the research compared to individual and microsystem levels, and although the research findings suggest that sport persistence is best explained by individual psychological factors, and that system levels, which are distanced from the individual, are becoming less prominent in persistent sport behaviour. Also, no replication or cross-validation of the clusters was attempted on a separate dataset yet which may provide a basis for further analysis.

## 5 Conclusions

The primary objective of the research was to explore the characteristics of sport persistence, the underlying factors and the determinants of its formation, which were specifically interpreted along the ecological model. Although our results are in line with previous research, the present study is unique in that no detailed analysis has yet been done in this specific approach. Our study not only confirms what has been known so far, but also provides a more accurate picture of the dynamics of the phenomenon in question, thus contributing to the advancement of the field. The experience and results of the present research allow us to create a sport persistence training that can effectively contribute to the performance and commitment of athletes, taking into account their individual strengths and weaknesses. This could provide sports professionals, coaches and psychologists with a toolbox to enhance athletes’ performance and also serve as a drop-out prevention tool. Such training programme can supports student-athletes’ perseverance in sport, with a focus on internal strengths, based on the research findings. Similar training programmes can be useful and effective when working with competitive athletes, for example to reduce drop-out tendencies, strengthen team relationships (37, 38). For recreational athletes, there is also the possibility of contextualising sporting support, which in an academic context could mean supporting secondary and tertiary studies through sport persistence (39).

## Data Availability

Data are available only on request due to ethical restrictions. For further information, please contact the following email address: kovacs.karolina@arts.unideb.hu.
