# Supplementary material for: Athlete profiles along grit, sport orientation and sport persistence based on a quantitative research on Hungarian athletes
Source: Front Sports Act Living. 2025 May 9;7:1594365. doi: 10.3389/fspor.2025.1594365 (PMC12098642; doi:10.3389/fspor.2025.1594365)
Supplement: Supplementary file 1 [file Table2.docx]

Appendix

**Table 1.** Distribution of athlete profiles by gender

|  | | **Gender** | | **Total** |
| --- | --- | --- | --- | --- |
|  |  | **Female** | **Male** |  |
| **disoriented endurance athletes** | N | 138 | 41 | 179 |
|  | Row% | 77.1% | 22.9% | 100.0% |
|  | Column % | 23.9% | 7.9% | 16.3% |
|  | Adjusted Residual | 7.1 | -7.1 |  |
| **dropout-prone athletes** | N | 112 | 59 | 171 |
|  | Row% | 65.5% | 34.5% | 100.0% |
|  | Column % | 19.4% | 11.4% | 15.6% |
|  | Adjusted Residual | 3.7 | -3.7 |  |
| **oriented lagging athletes** | N | 169 | 216 | 385 |
|  | Row% | 43.9% | 56.1% | 100.0% |
|  | Column % | 29.3% | 41.7% | 35.2% |
|  | Adjusted Residual | -4.3 | 4.3 |  |
| **consistently persistent athletes** | N | 158 | 202 | 360 |
|  | Row% | 43.9% | 56.1% | 100.0% |
|  | Column % | 27.4% | 39.0% | 32.9% |
|  | Adjusted Residual | -4.1 | 4.1 |  |
| **Total** | N | 577 | 518 | 1095 |
|  | Row% | 52.7% | 47.3% | 100.0% |
|  | Column % | 100.0% | 100.0% | 100.0% |

**Table 2.** Distribution of athlete profiles by the level of study

|  | | **Level of study** | | **Total** |
| --- | --- | --- | --- | --- |
|  |  | **Secondary** | **Tertiary** |  |
| **disoriented endurance athletes** | N | 51 | 129 | 180 |
|  | Row% | 28.3% | 71.7% | 100.0% |
|  | Column % | 9.6% | 22.6% | 16.3% |
|  | Adjusted Residual | -5.8 | 5.8 |  |
| **dropout-prone athletes** | N | 26 | 148 | 174 |
|  | Row% | 14.9% | 85.1% | 100.0% |
|  | Column % | 4.9% | 25.9% | 15.8% |
|  | Adjusted Residual | -9.6 | 9.6 |  |
| **oriented lagging athletes** | N | 193 | 194 | 387 |
|  | Row% | 49.9% | 50.1% | 100.0% |
|  | Column % | 36.3% | 33.9% | 35.1% |
|  | Adjusted Residual | 0.8 | -0.8 |  |
| **consistently persistent athletes** | N | 262 | 101 | 363 |
|  | Row% | 72.2% | 27.8% | 100.0% |
|  | Column % | 49.2% | 17.7% | 32.9% |
|  | Adjusted Residual | 11.2 | -11.2 |  |
| **Total** | N | 532 | 572 | 1104 |
|  | Row% | 48.2% | 51.8% | 100.0% |
|  | Column % | 100.0% | 100.0% | 100.0% |

**Table 3.** Distribution of athlete profiles by the father’s employment

|  | | **Labour market status of the father** | |  |
| --- | --- | --- | --- | --- |
|  |  | **unemployed** | **employed** |  |
| **disoriented endurance athletes** | N | 21 | 159 | 180 |
|  | Row% | 11.7% | 88.3% | 100.0% |
|  | Column % | 27.3% | 15.5% | 16.3% |
|  | Adjusted Residual | 2.7 | -2.7 |  |
| **dropout-prone athletes** | N | 23 | 151 | 174 |
|  | Row% | 13.2% | 86.8% | 100.0% |
|  | Column % | 29.9% | 14.7% | 15.8% |
|  | Adjusted Residual | 3.5 | -3.5 |  |
| **oriented lagging athletes** | N | 20 | 367 | 387 |
|  | Row% | 5.2% | 94.8% | 100.0% |
|  | Column % | 26.0% | 35.7% | 35.1% |
|  | Adjusted Residual | -1.7 | 1.7 |  |
| **consistently persistent athletes** | N | 13 | 350 | 363 |
|  | Row% | 3.6% | 96.4% | 100.0% |
|  | Column % | 16.9% | 34.1% | 32.9% |
|  | Adjusted Residual | -3.1 | 3.1 |  |
| **Total** | N | 77 | 1027 | 1104 |
|  | Row% | 7.0% | 93.0% | 100.0% |
|  | Column % | 100.0% | 100.0% | 100.0% |

**Table 4.** Distribution of athlete profiles by sporting frequency

|  | | Exercise frequency | | | | | | Total | |
| --- | --- | --- | --- | --- | --- | --- | --- | --- | --- |
|  |  | never | monthly | weekly | several times a week | per day | several times a day |  | |
| **disoriented endurance athletes** | N | 1 | 3 | 128 | 20 | 20 | 8 | 180 |  |
|  | Row% | 0.6% | 1.7% | 71.1% | 11.1% | 11.1% | 4.4% | 100.0% |  |
|  | Column % | 2.9% | 5.8% | 23.4% | 19.4% | 9.8% | 4.9% | 16.3% |  |
|  | Adjusted Residual | -2.2 | -2.1 | 6.3 | 0.9 | -2.8 | -4.3 |  |  |
| **dropout-prone athletes** | N | 21 | 22 | 76 | 34 | 15 | 6 | 174 |  |
|  | Row% | 12.1% | 12.6% | 43.7% | 19.5% | 8.6% | 3.4% | 100.0% |  |
|  | Column % | 60.0% | 42.3% | 13.9% | 33.0% | 7.4% | 3.7% | 15.8% |  |
|  | Adjusted Residual | 7.3 | 5.4 | -1.7 | 5.0 | -3.7 | -4.6 |  |  |
| **oriented lagging athletes** | N | 10 | 18 | 197 | 34 | 66 | 62 | 387 |  |
|  | Row% | 2.6% | 4.7% | 50.9% | 8.8% | 17.1% | 16.0% | 100.0% |  |
|  | Column % | 28.6% | 34.6% | 36.0% | 33.0% | 32.4% | 38.0% | 35.1% |  |
|  | Adjusted Residual | -0.8 | -0.1 | 0.7 | -0.5 | -0.9 | 0.9 |  |  |
| **consistently persistent athletes** | N | 3 | 9 | 146 | 15 | 103 | 87 | 363 |  |
|  | Row% | 0.8% | 2.5% | 40.2% | 4.1% | 28.4% | 24.0% | 100.0% |  |
|  | Column % | 8.6% | 17.3% | 26.7% | 14.6% | 50.5% | 53.4% | 32.9% |  |
|  | Adjusted Residual | -3.1 | -2.4 | -4.3 | -4.2 | 5.9 | 6.0 |  |  |
| **Total** | N | 35 | 52 | 547 | 103 | 204 | 163 | 1104 |  |
|  | Row% | 3.2% | 4.7% | 49.5% | 9.3% | 18.5% | 14.8% | 100.0% |  |
|  | Column % | 100.0% | 100.0% | 100.0% | 100.0% | 100.0% | 100.0% | 100.0% |  |

**Table 5.** Distribution of athlete profiles by the type of sport

|  | | **Type of sport** | |  |
| --- | --- | --- | --- | --- |
|  |  | **individual** | **team** |  |
| **disoriented endurance athletes** | N | 131 | 49 | 180 |
|  | Row% | 72.8% | 27.2% | 100.0% |
|  | Column % | 20.6% | 10.4% | 16.3% |
|  | Adjusted Residual | 4.5 | -4.5 |  |
| **dropout-prone athletes** | N | 145 | 29 | 174 |
|  | Row% | 83.3% | 16.7% | 100.0% |
|  | Column % | 22.8% | 6.2% | 15.8% |
|  | Adjusted Residual | 7.5 | -7.5 |  |
| **oriented lagging athletes** | N | 195 | 192 | 387 |
|  | Row% | 50.4% | 49.6% | 100.0% |
|  | Column % | 30.7% | 40.9% | 35.1% |
|  | Adjusted Residual | -3.5 | 3.5 |  |
| **consistently persistent athletes** | N | 164 | 199 | 363 |
|  | Row% | 45.2% | 54.8% | 100.0% |
|  | Column % | 25.8% | 42.4% | 32.9% |
|  | Adjusted Residual | -5.8 | 5.8 |  |
| **Total** | N | 635 | 469 | 1104 |
|  | Row% | 57.5% | 42.5% | 100.0% |
|  | Column % | 100.0% | 100.0% | 100.0% |

**Table 6.** Distribution of athlete profiles by sport level

|  | | **Sporting level** | |  |
| --- | --- | --- | --- | --- |
|  |  | **Recreational** | **Competitive** |  |
| **disoriented endurance athletes** | N | 129 | 51 | 180 |
|  | Row% | 71.7% | 28.3% | 100.0% |
|  | Column % | 22.6% | 9.6% | 16.3% |
|  | Adjusted Residual | 5.8 | -5.8 |  |
| **dropout-prone athletes** | N | 148 | 26 | 174 |
|  | Row% | 85.1% | 14.9% | 100.0% |
|  | Column % | 25.9% | 4.9% | 15.8% |
|  | Adjusted Residual | 9.6 | -9.6 |  |
| **oriented lagging athletes** | N | 194 | 193 | 387 |
|  | Row% | 50.1% | 49.9% | 100.0% |
|  | Column % | 33.9% | 36.3% | 35.1% |
|  | Adjusted Residual | -0.8 | 0.8 |  |
| **consistently persistent athletes** | N | 101 | 262 | 363 |
|  | Row% | 27.8% | 72.2% | 100.0% |
|  | Column % | 17.7% | 49.2% | 32.9% |
|  | Adjusted Residual | -11.2 | 11.2 |  |
| **Total** | N | 572 | 532 | 1104 |
|  | Row% | 51.8% | 48.2% | 100.0% |
|  | Column % | 100.0% | 100.0% | 100.0% |

**Table 7.** Distribution of athlete profiles by sports association membership

|  | | **Sports club membership** | |  |
| --- | --- | --- | --- | --- |
|  |  | **no** | **yes** |  |
| **disoriented endurance athletes** | N | 120 | 60 | 180 |
|  | Row% | 66.7% | 33.3% | 100.0% |
|  | Column % | 21.5% | 11.0% | 16.3% |
|  | Adjusted Residual | 4.8 | -4.8 |  |
| **dropout-prone athletes** | N | 138 | 36 | 174 |
|  | Row% | 79.3% | 20.7% | 100.0% |
|  | Column % | 24.8% | 6.6% | 15.8% |
|  | Adjusted Residual | 8.3 | -8.3 |  |
| **oriented lagging athletes** | N | 193 | 194 | 387 |
|  | Row% | 49.9% | 50.1% | 100.0% |
|  | Column % | 34.6% | 35.5% | 35.1% |
|  | Adjusted Residual | -0.3 | 0.3 |  |
| **consistently persistent athletes** | N | 106 | 257 | 363 |
|  | Row% | 29.2% | 70.8% | 100.0% |
|  | Column % | 19.0% | 47.0% | 32.9% |
|  | Adjusted Residual | -9.9 | 9.9 |  |
| **Total** | N | 557 | 547 | 1104 |
|  | Row% | 50.5% | 49.5% | 100.0% |
|  | Column % | 100.0% | 100.0% | 100.0% |
